# Effects of Fecal Microbiota Transplantation on Composition in Mice with CKD

**DOI:** 10.3390/toxins12120741

**Published:** 2020-11-24

**Authors:** Christophe Barba, Christophe O. Soulage, Gianvito Caggiano, Griet Glorieux, Denis Fouque, Laetitia Koppe

**Affiliations:** 1 CarMeN Lab, INSA-Lyon, INSERM U1060, INRA, University Claude Bernard Lyon 1, 69100 Villeurbanne, France; christophe.barba@chu-lyon.fr (C.B.); christophe.soulage@univ-lyon1.fr (C.O.S.); denis.fouque@chu-lyon.fr (D.F.); 2 Department of Nephrology, Hospices Civils de Lyon, Lyon Sud Hospital, 69310 Pierre Bénite, France; 3 Nephrology, Dialysis and Transplantation Unit, Department of Emergency and Organ Transplantation, University of Bari Aldo Moro, 70124 Bari, Italy; gianvitocaggiano@gmail.com; 4 Nephrology Section, Department of Internal Medicine and Pediatrics, Ghent University Hospital, 9000 Gent, Belgium; Griet.Glorieux@UGent.be

**Keywords:** chronic kidney disease, fecal microbiota transplantation, uremic toxins, p-cresyl-sulfate

## Abstract

Background: Chronic kidney disease (CKD) is a renal disorder characterized by the accumulation of uremic toxins with limited strategies to reduce their concentrations. A large amount of data supports the pivotal role of intestinal microbiota in CKD complications and as a major source of uremic toxins production. Here, we explored whether fecal microbiota transplantation (FMT) could be attenuated in metabolic complication and uremic toxin accumulation in mice with CKD. Methods: Kidney failure was chemically induced by a diet containing 0.25% (w/w) of adenine for four weeks. Mice were randomized into three groups: control, CKD and CKD + FMT groups. After four weeks, CKD mice underwent fecal microbiota transplantation (FMT) from healthy mice or phosphate buffered saline as control. The gut microbiota structure, uremic toxins plasmatic concentrations, and metabolic profiles were explored three weeks after transplantation. Results: Associated with the increase of alpha diversity, we observed a noticeable improvement of gut microbiota disturbance, after FMT treatment. FMT further decreased p-cresyl sulfate accumulation and improved glucose tolerance. There was no change in kidney function. Conclusions: These data indicate that FMT limited the accumulation of uremic toxins issued from intestinal cresol pathway by a beneficial effect on gut microbiota diversity. Further studies are needed to investigate the FMT efficiency, the timing and feces amount for the transplantation before, to become a therapeutic option in CKD patients.

## 1. Introduction

Kidney disease is one of the major health burdens worldwide, concerning approximately 350 million people worldwide and resulting in a high mortality rate of 1 million deaths/year among patients in a state of advanced chronic kidney disease (CKD) and end stage kidney disease (ESKD) [1]. Interestingly, in recent years, a growing body of data identified that dysbiosis in patients with CKD has been sculpting a detrimental metabolome, involved in detrimental clinical outcome [2]. The progression of CKD to ESKD and its cardiovascular complications are related to uremic toxins (UTs) accumulation [3]. A large proportion of these UTs originate from gut microbiota, such as indoxyl sulfate (IS) p-cresyl sulfate (pCS), and trimethylammine-N-oxide (TMAO) [4]. PCS and IS are mainly bound (~ 90–95%) plasma proteins (especially albumin) and therefore cannot be efficiently removed by conventional dialysis technic [5]. Up to date therapies for reducing production and levels of these UTs are missing but are needed to reduce morbi-mortality in patients with CKD [4,6,7].

Hence, therapies aimed at lowering the production of these bacterial toxins’ metabolites could be promising new therapeutic strategies. Recent studies suggested that removing the gut microbiota with antibiotics can significantly decrease the generation of several bacterial metabolites and UTs [6]; however, the administration of large-spectrum antibiotics with global activity is not acceptable as a long-term therapy. The modulation of intestinal microbiota with prebiotics or probiotics supplements is an attractive strategy, but at this time, results still remain controversial [7,8]. Indeed, without large randomized clinical trials investigating the effects of these strategies on hard clinical endpoints such as cardiovascular events or mortality, it is premature to recommend such interventions in routine clinical use. In this context, fecal microbiota transplantation (FMT), i.e., the reconstitution of the gut microbiota by transplantation of stool from a healthy volunteer, could offer a potent therapeutic approach in CKD [9]. The FMT was firstly validated in the management of *Clostridium difficile* infection. FMT is now considered a standard of care in the therapeutic management of recurrent *Clostridium difficile* infection [10]. Actually, FMT also showed promising results in other dysbiosis-associated disorders, including ulcerative colitis, metabolic syndrome and neurological disorders [11,12,13]. Regarding CKD, several publications demonstrated that the implantation of faecal microbiota from CKD patients into mice can reproduce insulin resistance and sarcopenia associated with uremia [14], increase UTs production and alter kidney function [2,3,4,5,6,7,8,9,10,11,12,13,14,15] It is important to note that in all these experimental settings, rodent models were either depleted of gut microbiota using broad spectrum antibiotics or germ-free models that limited the transposition to human study. Furthermore, the interpretation of experimental studies is sometimes difficult due to varying formulations of FMT and route of administration. Despite potential forcefulness of FMT, there are no supporting data concerning the application of FMT in CKD as therapeutic ambition, with the goal of correcting UTs generation and metabolic complications. 

Thus, the aim of this study was to determine the impact of FMT on circulating concentrations of bacterial metabolites (UTs) and metabolic complications associated with uremia in CKD mice models.

## 2. Results

### 2.1. Characteristics of the CKD Mice

**Figure 1:**
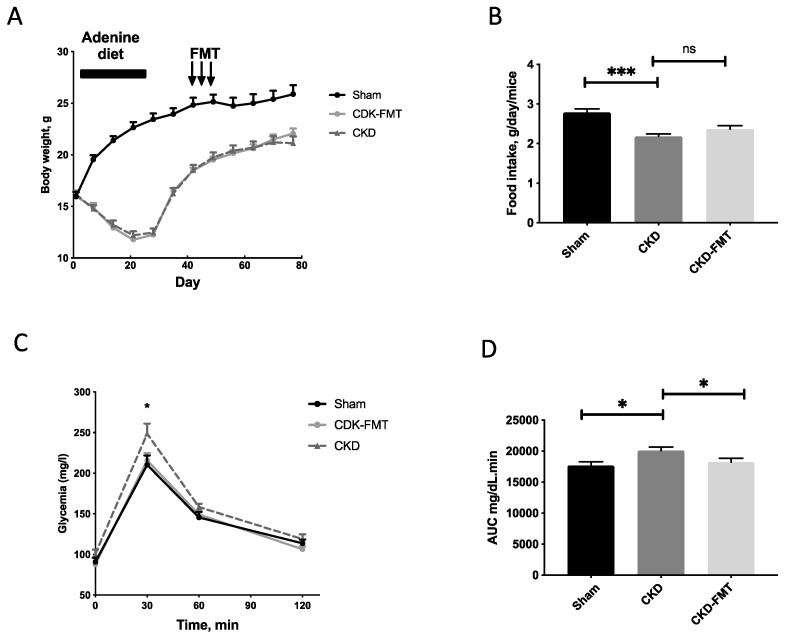
Body weight evolution and food intake, plasma glucose levels during glucose tolerance test. (**A**) Body weight evolution; Note that arrows indicate the sessions of fecal microbiota transplantation (FMT). (**B**) Daily food intake; (**C**) Blood glucose measured and (**D**) AUC during an i.p. glucose tolerance test (i.p.GTT, 1 g/kg D-glucose) in chronic kidney disease (CKD), CKD-FMT and control mice. * *p* < 0.05, *** *p* < 0.001 vs. CKD group; *n* = 9–10. (One-way ANOVA). Data are expressed as mean ± SEM for *n* = 9–10 in each group. Abbreviation: AUC: air under curve, FMT, fecal microbiota transplantation, ns: not significant.

**Figure 2:**
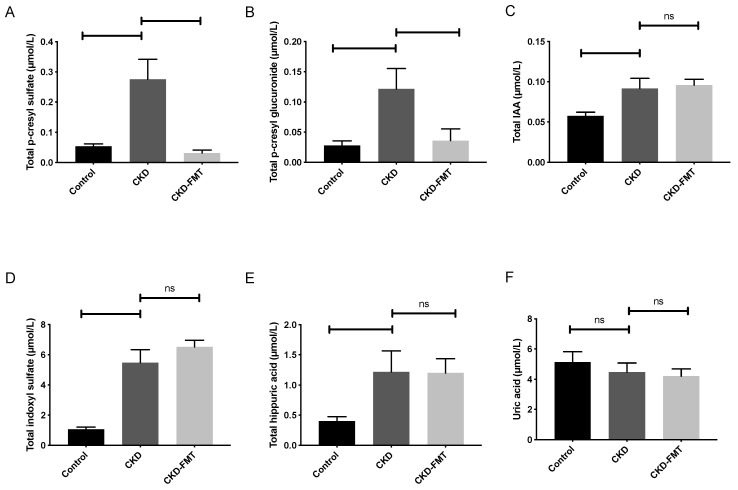
Effect of FMT on plasma levels of microbiota-derived uremic toxins. Plasma levels of p-cresyl sulfate (**A**), p-cresyl glucuronide (**B**), indole-3-acetic acid (**C**), indoxyl sulfate (**D**), hippuric acid (**E**) and uric acid (**F**). *n* = 9–10. vs. CKD group (ANOVA). Data are expressed as mean ± SEM for *n* = 9–10 in each group. Note that p-cresyl sulfate, p-cresyl glucuronide, indole-3-acetic acid and indoxyl sulfate are derived from gut microbiota, while hippuric acid and uric acid are produced by the host. Differences were considered to be significant at the *p* < 0.05 level (one-way ANOVA). Abbreviation: ns: not significant.

Figure 1 and Table 1 and Table 2 present data on food intake, body weight and several other biochemical parameters. Body weight and food intake were significantly decreased in both CKD groups compared with the control group, but no change was observed between CKD and CKD+FMT. (Figure 2A,B) As previously described [16], CKD was associated with glucose intolerance. FMT significantly improved glycemia levels during glucose challenge. (Figure 2C,D) Elevated blood creatinine and proteinuria levels were comparable between the CKD and CKD+ FMT (Table 2). We measured the gene expression of kidney fibrosis markers and pro-inflammatory cytokines, using real time PCR (Figure S1). Quantitative PCR indicated an increase of inflammatory markers and fibrosis in CKD mice compare to control mice. FMT did not prevent the expression of renal fibrosis-related genes (*TGFβ1* Transforming Growth Factor beta 1) and inflammatory cytokines (*IL-6, Interleukin 6 and TNFα, Tumor Necrosis Factor alpha)* in the kidney. In contrast, we observed a slight increase of *TGFβ1* expression after FMT in CKD mice compared to CKD mice, but we were not able to see any difference on fibrosis measured with Sirius-red positive areas on kidney histological sections. (Figure S2). Taken together, these results suggest that FMT did not significantly impaired or prevented the damage of renal function observed in CKD mice.

### 2.2. Effect of FMT on Microbiota-Derived Uremic Toxins

In order to investigate whether FMT could alter the accumulation of plasmatic UTs, we measured the total plasma levels of six representative UTs (Figure 2): p-cresyl glucuronide (PCG), p-cresyl sulfate (PCS), indole-3-acetic acid (IAA), indoxyl-sulfate (IS), hippuric acid (HA) and uric acid. Plasma PCS levels were 5.1-fold higher in the CKD than in the normal control group. (Figure 2A) FMT treatment in the CKD mice reduced the elevated plasma PCS levels (−88% compared with CKD mice receiving control solution; 0.28  ±  0.07 and 0.03 ± 0.01 µmol/L in the CKD and CKD+FMT groups, respectively, *p* < 0.01). Plasma PCG levels were 4.3-fold higher in the CKD group than in the control group. The elevated plasma PCG levels were also reduced by FMT (−71% compared with CKD mice receiving control solution; 0.12 ± 0.03 and 0.04 ± 0.02 µmol/L in the CKD and CKD+FMT, respectively, *p* < 0.05). (Figure 2B) In contrast, the elevated plasma of IS, IAA and HA levels in CKD mice were not modified by FMT treatment. (Figure 2C–E) Uric acid levels were not influenced by kidney function in mice and FMT treatment did not trigger significant changes in its plasma concentration. (Figure 2F)

### 2.3. Gut Effects of FMT on Microbiota Composition

**Figure 3:**
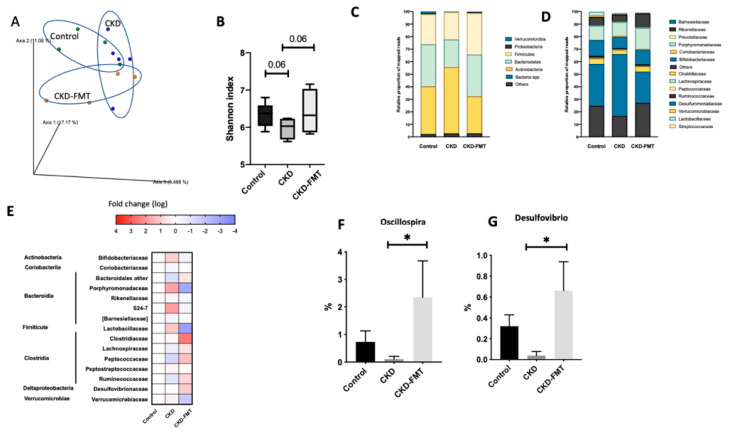
Effect of FMT on intestinal microbiota (**A**) Principal component analysis (PCA) of intestinal microbiota derived from CKD (blue dots), CKD + FMT-treated (orange dots) and control (green dots) mice. (**B**) Alfa diversity evaluated by the Shannon index. The Wilcoxon rank sum test was used to determine significance in α-diversity. Relative abundance of microbiota based on the average number of each subfamily at the phylum (**C**), and genus levels (**D**). (**E**) Heat map of the fold change of the indicated bacterial classes and families compare to control mice (**F**,**G**) The percentage of change in each subgroup at the genus level that significantly changed following FMT therapy. *n* = 4–5. * *p* < 0.05, vs. CKD group. (One-way ANOVA).

The principal component analysis (PCA) of the intestinal microbiome using weighted UniFrac showed differences between the three group: normal group and both CKD groups, even if there is still an overlap of the three groups (Figure 3A). A decrease in bacterial richness was shown in the CKD mice group that was totally restored by FMT. (Figure 3B) At the individual phylum level, Firmicutes, Bacteroidetes, and Actinobacteria were dominant in each of the three groups. Bacteroidetes tended to decrease in CKD mice and Firmicutes and Actinobacteria tended to increase in the CKD groups compared to control. (Figure 3C) At the family level, some minor populations of intestinal microbiota differed in the three group (Figure 3D,E). A more detailed classification at the genus level observed a trend of decrease in Oscillospira and Desulfovibrio in the CKD mice (Figure 3F,G). The decrease in Oscillospira and Desulfovibrio in CKD mice was abrogated by FMT treatment. These findings suggest that FMT treatment partly improved the dysbiosis in CKD mice.

## 3. Discussion

In this experimental study, we investigated the influence and effect of FMT on metabolic parameters, uremic toxin levels and fecal microbiota composition in CKD mice. We demonstrated that CKD led to a profound taxonomic and functional imbalance that could be reversed with an FMT from healthy mice. We demonstrated that FMT treatment successfully improved glucose tolerance on CKDmice and decreased plasma PCS and PCG accumulation and production. 

Individuals with CKD suffer from the toxin-induced uremic syndrome, a condition that severely compromises their survival and life quality and may require maintenance dialysis treatment. One reason for UTs accumulation in CKD patients is that some gut microbiota-derived uremic products cannot be removed by conventional dialysis. So, modulating intestinal microbiota by FMT might alleviate these devastating symptoms. In our model, we were able to reduce UTs production derived from tyrosine metabolism (PCS and PCG) but we did not observe any modification on UTs derived from tryptophan metabolism (IAA and IS). In a recent paper, Joossens et al. observed that ESKD patient with high levels of PCS or IS are associated with a significantly different intestinal microbiota composition [17]. To date, the bacteria responsible for indole and p-cresol and production have not been fully determined. Recently, data showed that phenolic compounds like PCS/PCG are mostly generated by anaerobic microbial species, whereas indolic compounds like IS/IAA are produced by both aerobes and anaerobes [18]. Some p cresol-producing bacteria in gut have been recently identified in previous studies including Clostridium difficile [19], Bilophila wadsworthia and Proteus vulgaris [2,20]. However, our experimental study failed to detect these species in the mice fecal samples, suggesting that other bacteria are also involved in the production of PCS and PCG. Similarly, in the literature, Alistipes shahii [2], Bacteroides thetaiotaomicron [20], Fusobacterium. Nucleatum [2], and Bacteroides ovatus [20] are indole-positive bacteria but we were not able to detect them. An increase in the Bacteroidetes-to-Firmicutes ratio and decrease in plasma UTs concentration were observed in CKD rats after a high fiber diet [21]. We did not observe any variation after FMT on the Bacteroidetes-to-Firmicutes ratio (data not shown), suggesting that the mechanisms and effects of FMT on the intestinal microbiota composition may differ from those of fibers and prebiotics. In CKD, a reduced richness of intestinal microbiome taxa and genes content has been observed in the majority of clinical and experimental studies [2,22]. In other pathologic situation, FMT in rodent was associated with increased bacterial richness [23]. Similarly, our experimental study has observed that FMT exerted a significant increase in alfa-diversity, which could explain, at least in part, the beneficial effects of FMT in our model. However, future research studies need to be performed to determine the signals and pathways connecting the protein/metabolite metabolism by the intestinal microbiota and host. 

Several studies have highlighted that FMT could restore the dysbiosis to promote host homeostasis [10,24]. In a previous study, we have demonstrated the impact of PCS on glucose metabolism and insulin sensitivity [16]. In good concordance, the reduction of PCS concentration by FMT in CKD mice is associated with the improvement of glucose intolerance. Our data support that FMT could be a putative therapeutic strategy to alleviate insulin resistance associated with uremia, which is associated with an increased risk of morbidity and mortality among CKD patients [16,25]. The modification of UTs levels and metabolite profiles cannot be explained by the modification of kidney function. Indeed, in our model, we were not able to demonstrate any beneficial effect of FMT on kidney function. These data are not surprising since FMT with feces from CKD mice did not alter renal function in normal germ-free mice [14]. Similarly, the reduction of UTs by other gut-targeted therapy such as AST 120 [26], or prebiotics [16] in CKD rodents were not sufficient to improve renal function. In addition, the duration of treatment in the present study was short (3 weeks) and we can hypothezise that the minor modification of UTs and glucose tolerance did not allow a significant improvement of kidney function. A long-term study is needed to validate this hypothesis.

Despite the large number of studies using FMT in animal models, the methods are highly debated. Actually, there are no scientific consensus and guidelines regarding the best technique and methodology. In addition, different practices in the preparation and the conservation of the inoculum can deeply influence FMT results. For instance, FMT may be transferred to recipient animal models immediately after collection, frozen without prior preparation, or frozen with a cryoprotectant [27]. The frequency and the length of inoculum administration range from a single administration to every day for one to several weeks. Germ-free and antibiotic-treated animals are commonly used for FMT studies, but issues with baseline values, reproducibility, and antibiotic resistance genes should be considered [28]. It is well known that antibiotic treatment may induce lesions by disturbing intestinal microbiota [29] and enriching antibiotic-resistant bacteria [30]. Furthermore, the use of antibiotics before FMT may be problematic when studying some pathology in rodent models, as they may be improved by antibiotics themselves. For example, antibiotic administration improved kidney ischemia-reperfusion damage by preventing the kidney inflammatory signaling and response [31]. Germ-free animals still seem to be the best controlled experimental model systems for FMT. However, the CKD model in germ-free mice provided more severe renal damage than in mice with commensal intestinal microbiota [32]. In contrast, ischemia-reperfusion damage was enhanced in germ-free mice when compared with conventional mice [33]. All these models cannot be applicated in human. Therefore, we have chosen to evaluate a FMT therapy based on conventional (i.e., not germ-free) mice without the use of antibiotics. It seems logical that pre-treatment with antibiotics might be a better treatment outcome in subject undergoing FMT. However, few experimental and clinical studies have addressed the contribution of antibiotic pre-conditioning before FMT to donor microbiota engraftment efficacy and results are still contradictory. For instance, Freitag et al., have observed that pre-treatment with antibiotics did not improve the engraftment of donor microbiota, but did improve the engraftment of specific bacteria in the context of Clostridium infection [34]. By contrast, Le Roy et al. observed that pre-treated groups (with laxatives or antibiotics) harbored a significantly better engraftment and phenotype in context of obesity [27]. These experimental strategies have not been documented in uremic conditions and we cannot exclude that the absence of modification of the concentration of the indole derivative was not related to the weakness of the microbiota implementation. Further studies are needed to explore different experimental conditions in order to improve the efficacy of FMT therapy in the context of CKD.

Our study has several limitations that deserve some comments. A first limitation could be represented by the adenine diet used for the induction of kidney failure. Indeed, Mishima et al. observed that adenine diet itself might modify the gut environment [32]. However, in a recent publication, Uchiyama et al. have observed that characteristics of gut microbiota in 5/6 nephrectomy mice were highly similar with those of adenine-fed mice. Indeed similar changes were observed in the relative abundance of each operational taxonomic unit at the phylum and class levels [14]. These results consolidate the fact that the uremic state, and not surgery or the adenine diet per se, induces dysbiosis. We however carefully respected a 3-week washout period between the adenine diet and the first FMT to allow a putative adenine induced dysbiosis to normalize. Another limitation is that the present study was only focused on a handful of UT concentrations and we cannot exclude the effects of other unanalyzed UTs, short-chain fatty acids (SCFA) or other gut metabolites. Our study is mainly descriptive as it was intended as a “proof of concept”. We did not provide any mechanistic insight into on the link between reduced UTS accumulation and improved glucose tolerance. We however, previously demonstrated that PCS can directly induce insulin resistance through activation of MAPKs (mitogen-activated protein kinases) [16] and thus, a reduction of PCS accumulation could per se improve glucose tolerance. We used PBS as control for FMT (rather than autologous feaces) and we cannot exclude the unspecific effect of the transplantation per se. Finally, microbiota composition and metabolic processes can be different in rodents compared to humans, which could limit the translational character of the data. Despite these limitations, we propose that uremic intestinal dysbiosis may be a novel therapeutic target and strategy against uremic complications in CKD patients.

## 4. Conclusions

In conclusion, the data described above suggest that FMT, associated to the other therapies such as dialysis, nutrition implementation and with a cocktail of relevant bacterial species, could be considered as a promising therapeutic strategy to restore the dysbiotic microbiota in CKD.

## 5. Materials and Methods 

### 5.1. Animals

**Figure 4:**
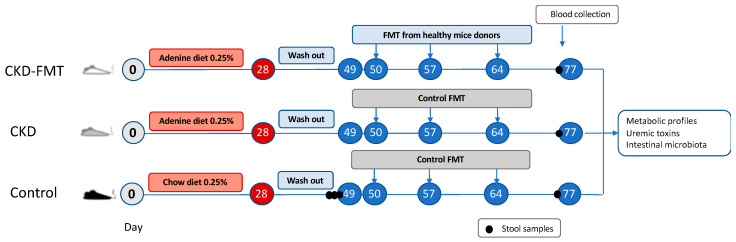
Experimental design of the study. C57BL/6J mice were fed with an adenine diet 0.25% for 4 weeks to induce chronic kidney disease (CKD). After 3 weeks of washout, mice were divided into 3 subgroups to receive either fecal microbiota transplantation (FMT) from control mice every week by oral gavage or Phosphate-Buffered Saline (PBS; referred to as control FMT) until terminal sacrifice after 3 weeks. Control group received only PBS.

Thirty male four-week-old male C57BL/6J mice were purchased from Janvier SA (Le Genest-Saint-Isle, France) and were group-housed in an air-conditioned room with a standardized environment of 21 ± 0.5 °C and 60–70% humidity with a 12 h light/dark cycle. Experimental design of the study is illustrated in Figure 4. Mice were randomly assigned in 3 groups and housed in 6 cages (5 mice per cage) in SPF condition. Mice were allowed a one-week period of acclimatization with free access to food and water. They were then assigned in two groups with 20 mice (CKD mice) receiving a 28 days adenine diet (0.25% w/w adenine on a A04 basis SAFE, Augy, France) to induce CKD, and 10 mice (control group) fed with a standard diet (SAFE, Augy, France). Body weight and food intake were measured once a week, and food intake was calculated for each cage as the difference between the amount given and that removed from the cage. All experimental procedures were performed in accordance with the guidelines laid down by the French Ministry of Agriculture (n°2013–118) and the European Union Council Directive for the protection of animals used for scientific purposes of September 22nd, 2010 (2010/63UE). The study protocol was approved by the local ethic committee (CETIL, Comité Ethique de l’INSA-Lyon, CNREEA n°102) on December 18th, 2018 under the reference #18109.

### 5.2. Fecal Microbiota Transplantation (FMT)

CKD mice were randomly assigned into two groups: (1) a group treated with FMT from healthy mice (CKD-FMT, *n* = 10) and (2) a group treated with vehicle solution (i.e., phosphate buffered saline, PBS) (CKD, *n* = 10). Control group (*n* = 10) was treated with vehicle solution (PBS). Feces from control mice were collected at the end of the wash out period in a clean cage, in the morning (8:00–10:00 a.m.). Feces from control mice were all pooled and mixed with sterile PBS (1 g of feces per 1 mL of PBS). The solution was vigorously and immediately mixed for 10 s before centrifugation at 800× *g* for 3 min. After centrifugation, the supernatant (fecal microbiota suspension) was stored in aliquot (300 µL) at −80 °C until it was used for FMT. Three weeks later, CKD-FMT mice were transplanted with fecal microbiota suspension (200 µL/mice) by gavage once per week for three weeks. The two other groups (control and CKD) were orally gaved with vehicle solution ((PBS, 200 µL/mice) according to the same pattern.

### 5.3. Diuresis and 24-h Proteinuria

After 3 weeks of FMT, mice were housed for 24 h by group of 3 into metabolic cages (Charles River laboratories, L’Abresle, France), to collect 24-h diuresis. Urine volume was determined gravimetrically, and protein concentration was evaluated according to the method of Bradford (Bradford reagent, Sigma Aldrich, Saint-Quentin Fallavier, France) using bovine serum albumin (BSA) as standard.

### 5.4. Euthanasia and Necropsy

At the end of the study, mice were anesthetized with isoflurane 4% and euthanized with sodium pentobarbital (400 mg/kg intraperitoneally, Doletal^®^). Body weight and body length were measured. Blood was drawn from cardiac puncture on heparinized syringe and centrifuged for 4 min at 3600× *g* to separate plasma. Plasma was collected, snap-frozen in liquid nitrogen and then stored at −80 °C until analysis. Caecum, kidneys, liver, heart, gastrocnemius muscle and two different pads of white adipose tissue (epididymal and retroperitoneal) were dissected out. All other tissues were weighted, snap-frozen in liquid nitrogen and stored at −80 °C.

### 5.5. Biochemical Analysis and Glucose Challenge

Plasma creatinine concentrations were measured with colorimetric assays from Cayman (Ann Arbor, MI, USA). For glucose tolerance test, after a 5-h fast, animals were injected intraperitoneally (i.p.) with 1 g/kg of D-glucose in sterile water. Blood glucose was measured prior to and 5, 10, 15, 30, 45, 60, 90, and 120 min after glucose injection. Blood glucose values were determined from a drop of blood sampled from the tail using an automatic glucose monitor (Accucheck performa, Roche, Meylan, France). The AUCs of blood glucose concentrations were calculated using GraphPad Prism software.

### 5.6. Analysis of Gene Expression

Kidney sample was crushed into liquid nitrogen, and total RNA was extracted using TRIzol Reagent, according to the manufacturer’s instructions (Life Technologies, Carlsbad, CA, USA). Real-time PCR assays were performed with Rotor-Gene 6000 (Qiagen) using SYBR qPCR Premix Ex Taq (Ozyme). TATA-box binding protein (TBP) was used as reference gene to normalize the results. Primers sequences are listed in Supplementary Materials (Table S1).

### 5.7. Renal Histology 

Kidneys were paraffin-embedded and stained using Sirius red staining (Cellimaps, Dijon, France). Pictures of non-overlapping fields were taken with an Olympus microscope. We used Sirius red staining to quantify the area corresponding to collagen fibrils for each image. Fibrosis area was measured using ImageJ software (https://imagej.nih.gov/ij/).

### 5.8. Measurement of Uremic Toxins

Total concentrations of UTs were quantified by ultra-high performance liquid chromatography coupled with fluorescence detection (UPLC-FLD) as previously described [35]. 

### 5.9. Fecal Microbiota Analysis by 16S rRNA Gene Sequencing

The genomic DNA of gut microbiota was extracted from murine cecal contents, and 16S rRNA genes in the DNA samples were analyzed using a MiSeq sequencer (Illumina, San Diego, CA, USA) Microbiome analysis were performed by Biomnigene company (www.biomnigene.fr, Besançon, France), as previously reported [36]. Briefly, microbial genomic DNA was extracted using the E.Z.N.A Stool DNA kit (Omega Bio-tek, Norcross, GA, USA) following the manufacturer’s instructions. The V3-V4 hyper-variable region of the 16S rRNA gene was amplified by using the following primers: forward primer 5′-CCTACGGGNGGCWGCAG-3′ and reverse primer 5′-GGACTACHVGGGTWTCTAAT-3′, in which bases indexes were incorporated to perform multiplexing. The PCR reactions were performed using 10 ng of fecal DNA, 0.4 μmol/L primers, 200 μmol/L dNTP, and 1X of PrimeSTAR^®^ Max DNA Polymerase (Takara Bio Europe, Saint-Germain-en-Laye, France). Amplifications were carried out using the following program: 1 cycle at 98 °C for 5 min, followed by 37 cycles at 98 °C for 10 s, 58 °C for 15 s, 72 °C for 5 s, and finishing with a step at 72 °C for 1 min. The PCR products were analyzed on Qiaxcel Cartridge (QIAGEN, Courtaboeuf, France) to verify the amplification. For the preparation of the libraries, the concentration of the PCR was determined by fluorometric assay on Qubit™ 4.0 (dsDNA HS aAsay kit—ThermoFisher) according to manufacturer’s recommendations, in order to determine the precise molarity of PCR products for each sample; the samples were grouped in an equimolar manner. The pool of PCR products was then purified by electrophoresis on PippinHT using a 1.5% agarose cassette (Sage Sciences, Beverly, MA, USA). The sequencing of V3-V4 amplicons was carried out on MiSeq Illumina in 2X251 bp by using the Illumina MiSeq Reagent Kit v2 (500 cycles; Illumina, San Diego, CA, USA).

### 5.10. Statistical Analysis

Data are expressed as mean ± SEM. Statistical analyses were performed using GraphPad Prism version 8.0 (GraphPad Software, La Jolla, CA, USA). The D’Agostino and Pearson test was used to assess normality. Student t test or Mann–Whitney U test were used for simple comparison, whereas ANOVA or Kruskal–Wallis analyses were performed for multiple comparisons. Welch’s correction was applied in the case of variance inhomogeneity. Alpha diversity (within sample diversity) was calculated using Shannon’s metrics as implemented in QIIME [37]. The Wilcoxon rank sum test was used to determine significance in α-diversity. Beta diversity was assessed using unweighted UniFrac distances. Principal coordinates analysis (PCoA) was performed by QIIME to visualize the dissimilarity matrix (beta-diversity) between all the samples. Moreover, 3D principal component analysis (PCA) plots were generated using EMPEROR (https://github.com/biocore/emperor). Multiple post-hoc comparisons using Tukey test and Dunnett correction were performed to identify significant differences between groups. A *p* value < 0.05 was considered as statistically significant.

## Figures and Tables

**Table 1:** Biometric data.

Variable	Control			CKD			CKD-FMT		
*n*	10			10			9		
Biometric data									
BW (g)	26	±	1^a^	21	±	1^b^	22	±	1^b^
BL (cm)	9.5	±	0.1^a^	8.9	±	0.1^b^	8.9	±	0.1^b^
Lee index (×10^3^)	312	±	2^a^	312	±	2^a^	317	±	4^a^
Adipose tissue weight									
Intra-abdoWAT (mg/10 g BW)	167	±	14^a^	130	±	9^a^	131	±	5^a^
rWAT (mg/10 g BW)	36	±	5^a^	18	±	2^b^	22	±	1^b,c^
eWAT (mg/10 g BW)	131	±	9^a^	112	±	8^ab^	70	±	17^b^
Organ weight									
Heart (mg/10 g BW)	50	±	2^a^	54	±	1^a^	53	±	1^a^
Gastrocnemius (mg/10 g BW)	56	±	3^a^	56	±	2^a^	55	±	1^a^
Liver (mg/10 g BW)	452	±	8^a^	405	±	8^b^	467	±	9^a^
Kidneys (mg/10 g BW)	114	±	3^a^	77	±	1^b^	78	±	2^b^

Data are expressed as mean ± SEM. Abbreviations: CKD, chronic kidney disease, FMT, fecal microbiota transplantation, BW, body weight, BL, body length, WAT, white adipose tissue, Intra-abdoWAT: intra-abdominal WAT, rWAT, retroperitoneal WAT, eWAT, epididymal WAT. Adipose tissue and organ weights are expressed in mg/10 g body weight. Lee index, a common index of adiposity in rodent, was calculated as the cubic square of body weight divided by naso-anal length. Different letters indicate a significant difference at the *p* < 0.05 level (one-way ANOVA).

**Table 2:** Biochemical data.

Variable *n*	Control			CKD			CKD-FMT		
	10			10			9		
Fasting glucose (mmol/L)	5.0	±	0.3^a^	5.5	±	0.3^a^	4.8	±	0.2^a^
Serum creatinine (µmol/L)	8.52	±	0.4^a^	16.0	±	1.1^b^	17.2	±	0.5^b^
Urinary output (mL/24 h)	0.3	±	0.1^a^	2.1	±	0.4^a^	1.9	±	0.8^a^
Urinary protein (mg/24 h)	0.5	±	0.2^a^	0.5	±	0.2^a^	0.6	±	0.3^a^

Data are expressed as mean ± SEM. Abbreviations: CKD, chronic kidney disease, FMT, fecal microbiota transplantation. Different letters indicate a significant difference at the *p* < 0.05 level (one-way ANOVA).

## Supplementary Materials

The following are available online at https://www.mdpi.com/2072-6651/12/12/741/s1. Figure S1: Gene expression of fibrosis and pro-inflammatory markers in kidney. Figure S2: Evaluation of kidney fibrosis in CKD mice with and without FMT. Table S1. Sequence of primers used for qPCR analysis.
