# Supplementary material for: Effects of Fecal Microbiota Transplantation on Composition in Mice with CKD
Source: Toxins (Basel). 2020 Nov 24;12(12):741. doi: 10.3390/toxins12120741 (PMC7761367; doi:10.3390/toxins12120741)
Supplement: Supplementary file 1 [file toxins-12-00741-s001.pdf]

Supplementary Materials: Effects of Fecal Microbiota Transplantation on Composition in Mice with CKD

Christophe Barba, Christophe O. Soulage, Gianvito Caggiano, Griet Glorieux, Denis Fouque and Laetitia Koppe

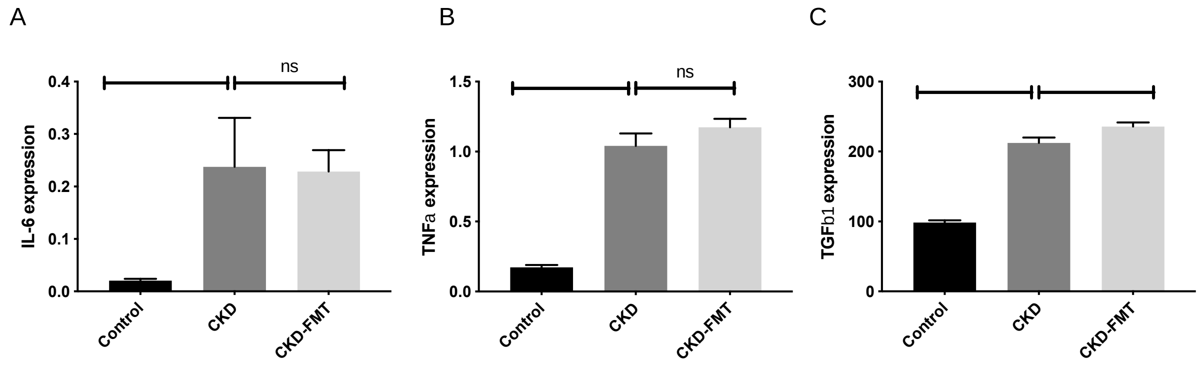

Figure S1. Gene expression of fibrosis and pro-inflammatory markers in kidney.

Effects of FMT on relative mRNA expression of (**A**) IL-6 (Interleukin 6), (**B**) TNFα (Tumor Necrosis Factor alpha) and (**C**) TGFβ1 (Transforming Growth Factor beta 1). TBP (TATA-Box Binding Protein) was used as reference gene to normalize the results. Data are expressed as mean ± SEM for n = 7–10 animals in each group. *$p < 0.05$, ***$p < 0.001$ vs CKD; (ANOVA and Dunnett post hoc test).

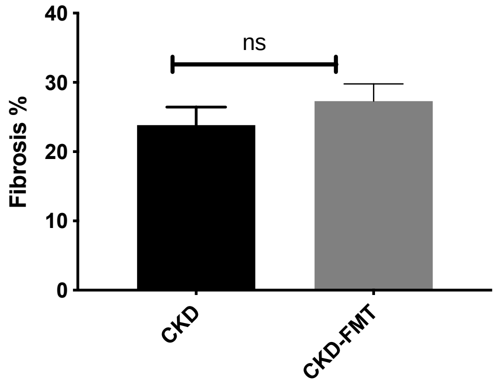

Figure S2. Evaluation of kidney fibrosis in CKD mice with and without FMT.

Sirius red morphometric evaluation in CKD mice treated with and without FMT. CKD: chronic kidney disease. FMT: Feacal microbiota transplantation. Data are expressed as mean ± SEM for n = 8-10 animals in each group. T-test.

Table S1. Sequence of primers used for qPCR analysis.

Gene name	Gene ID	Forward Primer	Reverse Primer
Tgfb1	21803	AGGGCTACCATGCCAACTTC	GTAACTGAGTTCTGACAGTG
TNF alpha	21926	CCAGACCCTCACACTCAGATC	CACTTGGTGGTTTGCTACGAC
IL6	16193	AGTTGCCTTCTTGGGACTGAT	TCCACGATTTCCCAGAGAAC
TBP	21374	TGGTGTGCACAGGAGCCAAG	TTCACATCACAGCTCCCCAC

DNA sequences of primers used for qPCR analysis. Abbreviations: qPCR: Quantitative Polymerase Chain Reaction, Tgfb1: Transforming Growth Factor beta 1, TNF alpha: Tumor Necrosis Factor alpha, IL-6: Interleukin 6, TBP: TATA-box binding protein.
